# Overlooked Leadership Potential: The Preference for Leadership Potential in Job Candidates Who Are Men vs. Women

**DOI:** 10.3389/fpsyg.2019.00755

**Published:** 2019-04-16

**Authors:** Abigail Player, Georgina Randsley de Moura, Ana C. Leite, Dominic Abrams, Fatima Tresh

**Affiliations:** Centre for the Study of Group Processes, School of Psychology, University of Kent, Canterbury, United Kingdom

**Keywords:** leadership, potential, gender, women, talent management, hiring decision

## Abstract

Two experiments tested the value people attach to the leadership potential and leadership performance of female and male candidates for leadership positions in an organizational hiring simulation. In both experiments, participants (*Total N* = 297) valued leadership potential more highly than leadership performance, but only for male candidates. By contrast, female candidates were preferred when they demonstrated leadership performance over leadership potential. The findings reveal an *overlooked potential effect* that exclusively benefits men and hinders women who pursue leadership positions that require leadership potential. Implications for the representation of women in leadership positions and directions for future research are discussed.

## Introduction

“Women hold up more than half the sky and represent much of the world’s unrealized potential.” Ki Moon (2011)

The unbalanced representation of women in leadership is a significant social, cultural, and organizational issue. Given that women now represent 40% of the global working population (The World Bank, 2017), it would be reasonable to expect a comparable gender ratio in leadership roles. However, women only represent 34% of managerial positions around the world (World Economic Forum [WEF], 2018), and even less in the top roles. For example, in the United States less than 5% Fortune 500 CEOs are women (Zarya, 2018). Thus, the persistent underrepresentation of female CEOs across different countries suggests that women face significant gender bias in the processes involved in the hiring and promotion of leaders. It may be that women’s different career trajectories render them less likely to occupy management positions than men (e.g., Eagly and Karau, 1991, 2002; Ryan and Branscombe, 2012; Hoobler et al., 2014). Moreover, some research indicates that there are exceptions to the preferential selection of male leaders, with women more likely to be appointed to risky or precarious positions for example (*glass cliff*, see Ryan and Haslam, 2005). Nonetheless, the evidence overall indicates that women are less likely than men to be appointed to top leadership roles (Moss-Racusin et al., 2012; Chartered Management Institute [CMI], 2016; Glass and Cook, 2016).

### Leadership Potential

Identifying talent for the future is key for organizations, and confers a competitive advantage (Silzer and Dowell, 2010). Talent management systems and leadership potential programs are designed to identify those individuals who will be leaders in the future and occupy senior positions (Church et al., 2015). *Leadership potential* specifically refers to exhibiting the qualities that signal future leadership effectiveness (e.g., Silzer and Borman, 2017). There are several frameworks that identify key characteristics of leadership potential, one of the most prominent being analytical capability (e.g., strategic insight, Dries and Pepermans, 2012). However, most research on leadership potential has confounded it with current and past performance rather than on distinct indicators of leadership potential (Silzer and Church, 2009). Specifically, leadership potential and leadership performance are highly conflated in practice, because indicators of high performance often provide the only source of information about potential. The use of high-performance indicators to measure potential has been criticized because performance is limited to the requirements of an individual’s current role, and may not extend to success at the next level (Robinson et al., 2009). Indeed, performance indicators can create a “halo effect” that may overinflate perceptions of leadership potential (Balzer and Sulsky, 1992; Konczak and Foster, 2009).

An operational distinction between potential and performance was provided by Tormala et al. (2012). Participants were presented with competing candidates who were either higher in potential or higher in performance. Future potential overshadowed previous performance with respect to participants’ evaluations of impressiveness and endorsement across a range of domains (e.g., art, sport, graduate school entry, and job recruitment). For example, participants judged two candidates with equivalent educational and professional backgrounds for a managerial position at a large company (Tormala et al., 2012, Experiment 2). One of the candidates had purportedly scored higher on a *leadership achievement inventory*, whereas the other scored higher on an *assessment of leadership potential*. Participants recognized that the candidate with higher leadership achievement had a more impressive résumé, but they expected the candidate with higher leadership potential to perform better in the future. Therefore, in this research we operationalize leadership potential and leadership performance as distinct leadership characteristics.

Assessments of leadership performance involve judgments of a number of different leadership traits or characteristics (e.g., vision, interpersonal, task-orientated). Previous research has found that assessments of women were higher than those of men on leadership performance but lower than those of men on vision and strategy (e.g., Ibarra and Obodaru, 2009; Roth et al., 2012). Differential ratings on vision and strategy might have consequences for leadership selection given that strategic insight, and analytical skills in general, are acknowledged as key indicators of leadership potential (e.g., Marshall-Mies et al., 2000; Silzer and Church, 2009; Dries and Pepermans, 2012). For example, Ibarra and Obodaru (2009) studied 2,816 female and male executives across 149 countries, analyzing 22,244 evaluations, and found that women were rated better than or equal to men across a range of measures but that men were rated significantly higher than women on “visioning” – the ability to be able to put forward a compelling vision and strategy. Moreover, a meta-analysis of field studies (*N* = 45,733) revealed that women were evaluated more favorably than men on overall job performance ratings. Yet women were rated lower than men on the measure of *future* performance and *promotability* (Roth et al., 2012). Such differences might arise partly because women are more likely to take on tasks which require competence, but do not improve chances of promotion (e.g., committee service; Babcock et al., 2017). Nonetheless, research on the power of gender stereotypes and decisions about leadership is conclusive – all else being equal, women are judged more harshly than men (e.g., Rudman and Glick, 2001; Lyness and Heilman, 2006; Blau and DeVaro, 2007).

### Gender Bias in Leadership Selection

Social roles include both descriptive beliefs that define what men and women are like, and also prescriptive norms that define how individuals should be and how they should not be (Eagly et al., 2000; Eagly and Wood, 2012). According to social role theory (Eagly and Wood, 1999, 2012), typical gender roles (e.g., women overpopulating communally demanding roles and men overpopulating agentically demanding roles) are likely to persist because people consistently witness typically female and male behavior and conclude that these characteristics are representative of the sexes. Indeed, because people are frequently exposed to typical sex-typed behavior, women are typically perceived as, and expected to be, *communal* (e.g., caring, sensitive), whereas men are expected to be *agentic* (e.g., determined, competitive; Eagly and Karau, 1991; Eagly et al., 1995; Heilman, 2001; Eagly and Sczesny, 2009; Rosette and Tost, 2010; Koenig et al., 2011). In those workplaces where agency instead of communality is expected, stereotypes produce distinctive penalties for women (Caleo and Heilman, 2013). In particular, meta-analysis shows that leadership roles are still typically viewed as being agentic (Koenig et al., 2011), and therefore men will be perceived as more capable leaders (Levinson and Young, 2010).

When women demonstrate success in leadership roles, they can be penalized because they violate gender-prescriptive norms (Heilman et al., 2004) or contextual expectations (e.g., Randsley de Moura et al., 2018). Ultimately, when people interrupt gender stereotypes, they can suffer consequences that undermine and devalue their social and economic status (Rudman and Phelan, 2008). Women who put themselves forward for positions of leadership can therefore face backlashes that undermine their status (Rudman and Phelan, 2008). In support of this idea, the devaluation of women leaders is more pronounced when they occupy male-dominated roles (Eagly et al., 1992). Meta-analysis has also highlighted that women who display explicitly dominant behaviors (e.g., direct demands) are perceived as less hirable – because they are rated lower in likeability rather than any reduction in perceived competence (Williams and Tiedens, 2016).

In this paper, we test the hypothesis that women’s leadership potential is more likely to be dismissed than men’s leadership potential. This is consistent with the “think manager-think male” phenomenon (e.g., Schein et al., 1996). Substantial evidence suggests that the stereotype of a typical leader is highly congruent with masculine traits (Eagly and Karau, 2002; Koenig et al., 2011). The incongruence between the stereotype of a typical leader and feminine traits may explain why women face more challenging thresholds for promotion. For example, Lyness and Heilman (2006) found that women who occupied management positions that were typically characterized by organizational power and influence (i.e., gender role incongruous) also received lower performance ratings than their male counterparts. In summary, we expect an *overlooked potential effect* such that women’s but not men’s leadership potential is likely to be overlooked when people judge and select candidates for leadership.

Although research indicates that evaluations of leaders and promotion to leadership positions are likely to be biased in favor of men, a meta-analysis (Koch et al., 2015; *N* = 22,348) revealed a bias for men in male-dominated roles (e.g., in a leader position). However, that role congruity bias was attenuated when information clearly highlighted a candidate’s high competence. We hypothesized that a female candidate’s leadership potential may only be acknowledged if she is unambiguously a high performer (i.e., when her leadership achievements cannot be dismissed).

## Overview of Studies

Previous studies have found that gender role incongruity (see Heilman and Eagly, 2008) contributes to gender inequality in leadership positions, but to date there is no explicit experimental evidence on gender biases in the recognition of leadership potential. Given the importance of recognizing and effectively managing talent for businesses (Church, 2014), it is essential to investigate gender as a boundary condition to perceptions of leadership potential. Holding constant the actual traits and performance of candidates, two experimental studies used simulated hiring decisions to investigate whether leadership potential is overlooked in women, but not in men.

We used a simulation of organizational hiring of candidates applying for leadership positions. This experimental vignette methodology was used as it is regarded as a reliable and accurate method that allows greater control of the research process (Handley et al., 2007; Doz, 2011). In addition, we recruited participants through online crowdsourcing portals to provide relevant samples (e.g., Buhrmester et al., 2011; Holden et al., 2013).

Experiment 1 tested the effects of candidate gender on the recognition of leadership potential. Specifically, we tested whether there is a preference for potential in both male and female candidates, or whether people overlook leadership potential in female candidates. We also explored whether the decision makers’ gender moderated the preference for potential in each gender of candidate. Experiment 2 investigated the evaluation of leadership potential and leadership performance, candidate gender, and decision makers’ gender when leaders were being hired for a senior management position. Taken together, these studies examined whether leadership potential is overlooked in women who seek progression into leadership positions, relative to men with identical résumés.

Specifically, we hypothesized that participants would prefer leadership potential over leadership performance (Hypothesis 1). We expected that participants would prefer leadership potential more in male candidates than in female candidates (Hypothesis 2). More importantly, when it comes to candidate choice, we hypothesized that participants would prefer leadership potential over leadership performance in male candidates (Hypothesis 3); but leadership performance over leadership potential in female candidates (Hypothesis 4). In addition, we hypothesized that high leadership potential male candidates would be selected more than high potential female candidates (Hypothesis 5).

All experiments were carried out in accordance with the recommendations of the School of Psychology Ethics Committee at the University of Kent, United Kingdom. The protocol was approved using the School of Psychology Ethics system. All participants gave written informed consent in accordance with the Declaration of Helsinki. The research was conducted in accordance with guidelines from the University of Kent Research Ethics (Human Participants) Committee, the Economic and Social Research Council (ESRC) Research Ethics Framework, and the ethical guidelines from the British Psychological Society (BPS).

## Experiment 1

### Materials and Methods

#### Participants and Design

We recruited 98 participants (59 males and 39 females, *M*_age_ = 36.38, 79.6% employed) via *Amazon MTurk*. The quasi-experimental design was a 2 (Leadership Characteristic: leadership potential, leadership performance) × 2 (Candidate Gender: female, male) × 2 (Participant Gender: female, male) mixed design, with leadership characteristic and candidate gender as within-participant factors. All additional candidate information (e.g., age, qualifications, work experience, GPA) was counterbalanced.

#### Procedure and Materials

Participants were presented simultaneously with four candidates (male candidate with leadership potential, male candidate with leadership performance, female candidate with leadership potential, female candidate with leadership performance; see Appendix in random order from left to right). Participants were asked to imagine they worked for a hypothetical organization “ALPHATech” and that they were involved in the recruitment and selection of a new employee:

“*ALPHATech is a successful business providing financial and economic advice (e.g., tax, investments, account management, and pensions) to a number of different industries. Imagine that you work in a human resources role and you are part of the team responsible for recruiting and hiring new employees. ALPHATech are currently expanding their business and as part of this are recruiting for a number of positions within the company. Imagine that you are part of the hiring panel and you have been given some candidates to evaluate.”*

Candidate potential and performance were manipulated by adjusting the score on two assessments: leadership achievement and leadership potential. Specifically, as in Tormala et al. (2012, Experiment 2), the Leadership Achievement Inventory manipulated a high or moderate performer by varying the score (83/100 or 96/100) and the accompanying paragraphs as follows:

“The LAI gauges leadership achievement, defined as an individual’s observed (i.e., actual) leadership performance at the current stage in his or her career. An achievement score of 83 places this applicant in the top 17% of people who have been assessed [An achievement score of 96 places this applicant in the top 4% of people who have been assessed].

The Assessment of Leadership Potential score was accompanied by the following paragraph^1^, which varied depending on the condition (high or moderate leadership potential):

“The ALP gauges leadership potential, defined as the employee’s predicted leadership performance in the near future. A score of 96 indicates that this applicant predicted future leadership performance is estimated to be in the top 4% of people who have been assessed [A score of 83 indicates that this applicant predicted future leadership performance is estimated to be in the top 17% of people who have been assessed].”

Thus, in the leadership potential condition, the applicant had received a higher score on potential (top 4%) and a more moderate score (top 17%) on leadership achievement, whereas in the leadership performance condition, the applicant had received a moderate leadership potential score (top 17%) and a high performance score (top 4%). High and moderate scores were used rather than high and low scores, in order to focus attention on the dimension at which the candidate excelled rather than suggesting any weakness (see Appendix). The focus on leadership potential or leadership performance was reinforced through comments ostensibly taken from a panel review, for example:

“*This candidate has great prospects. She has some exciting new ideas for the future of the team and the organization, which could offer the opportunity to increase sales and performance in the future.”* [Leadership Potential]

“*The applicant is highly capable, and has consistently performed above his own objectives and that of the organizations. The performance in his current role has exceeded expectations.”* [Leadership Performance]

### Measures

#### Candidate Hiring

Candidate hiring was measured using two items on a 9-point rating scale (α = 0.78): “How interested would you be in hiring each applicant?,” “To what extent do you think hiring each applicant would be a good decision or a bad one?” Lower values indicate less hiring intention.

#### Expected Success

Expected success was measured using one item asking participants “How successful do you think each applicant will be in their career?” (1 – *not at all successful*, 9 – *very successful*).

#### Résumé Evaluation

Résumé evaluation was measured by asking participants to compare all four applicants and decide “in your opinion, which applicant has the most impressive résumé?” They were required to rank candidates from *first* (most impressive) to *fourth* (least impressive).^2^

#### Future Performance

Future performance was measured with an order of preference based on performance, “which applicant do you think will perform better by the fifth year at ALPHATech?” Candidates were ranked from best future performance (*first*) to worst future performance (*fourth*).

### Results

We conducted a Leadership Characteristic (leadership potential, leadership performance) × Candidate Gender (female, male) × Participant Gender (female, male) mixed ANOVA to analyze the evaluation items of candidate hiring and expected success. We hypothesized that participants would be more willing to hire candidates with leadership potential and would expect those candidates to be more successful than candidates with leadership performance (Hypothesis 1). Furthermore, we expected these effects to be stronger for male candidates (Hypothesis 2 and Hypothesis 3). We did not hypothesize participant gender effects but included this factor as exploratory.

Friedman tests and Wilcoxon Signed Ranks tests were used to analyze whether there were differences in the choice-based rankings of each candidate’s résumé and expected future performance. We expected participants to rank the male candidate with leadership potential higher than the male candidate with leadership performance on the evaluation of résumés and expected future performance. We expected the opposite pattern for female candidates (Hypothesis 4). Finally, we expected participants to rank the male candidate with leadership potential higher than the female candidate with leadership potential in both the evaluation of résumés and expected future performance (Hypothesis 5).

#### Candidate Hiring

There was a significant main effect of candidate gender, *F*(1,96) = 5.15, *p* = 0.025, η^2^ = 0.05, with female candidates rated as more likely to be a good hire than male candidates, see Table 1. The main effect of leadership characteristic was non-significant, *F*(1,96) = 1.40, *p* = 0.240, η^2^ = 0.01, as was the main effect of participant gender, *F*(1,96) = 0.42, *p* = 0.838, η^2^ < 0.001. There was a significant Candidate Gender × Participant Gender interaction, *F*(1,96) = 9.77, *p* = 0.002, η^2^ = 0.09. Simple main effects of candidate gender within levels of participant gender were analyzed. There was a significant difference for female participants’ evaluation of male and female candidates, *F*(1,96) = 12.09, *p* = 0.001, η^2^ = 0.11, who expressed a preference for female candidates over male candidates overall (Table 1). This was not hypothesized, but demonstrates ingroup bias for female participants. There was no significant difference among male participants, *F*(1,96) = 0.46, *p* = 0.499, η^2^ = 0.01. There were no simple main effects of participant gender within level of candidate gender. Female and male participants did not make significantly different hiring evaluations of female candidates, *F*(1,96) = 3.59, *p* = 0.06, η^2^ = 0.04, or of male candidates, *F*(1,96) = 1.88, *p* = 0.174, η^2^ = 0.02. There was no significant Leadership Characteristic × Candidate Gender × Participant Gender interaction effect, *F*(1,96) = 1.69, *p* = 0.196, η^2^ = 0.017.

**Table 1:** Means and standard errors by candidate gender, participant gender, and leadership characteristic for candidate hiring and expected success (Experiment 1).

	Participant gender	Dependent measure	Leadership potential	Leadership performance	Overall
Female candidate	Female	Candidate hiring	7.55 (0.20)	7.60 (0.22)	7.58 (0.17)
		Expected success	7.74 (0.18)	7.97 (0.23)	7.86 (0.18)
	Male	Candidate hiring	7.20 (0.16)	7.13 (0.18)	7.17 (0.14)
		Expected success	7.48 (0.15)	7.24 (0.18)	7.36 (0.14)
	Overall	Candidate hiring	7.38 (0.13)	7.37 (0.14)	7.37 (0.11)
		Expected success	7.61 (0.12)	7.61 (0.15)	7.61 (0.11)
	Female	Candidate hiring	7.21 (0.20)	6.67 (0.27)	6.94 (0.19)
		Expected success	7.87 (0.17)	7.23 (0.28)	7.55 (0.18)
Male candidate	Male	Candidate hiring	7.31 (0.16)	7.23 (0.22)	7.27 (0.15)
		Expected success	7.51 (0.14)	7.34 (0.23)	7.42 (0.15)
	Overall	Candidate hiring	7.26 (0.13)	6.95 (0.17)	7.10 (0.12)
		Expected success	7.69 (0.11)	7.29 (0.18)	7.49 (0.12)
	Female	Candidate hiring	7.38 (0.17)	7.14 (0.20)	7.16 (0.15)
		Expected success	7.81 (0.16)	7.60 (0.22)	7.71 (0.16)
Candidate gender collapsed	Male	Candidate hiring	7.25 (0.14)	7.18 (0.16)	7.22 (0.12)
		Expected success	7.49 (0.13)	7.29 (0.18)	7.39 (0.13)
	Overall	Candidate hiring	7.32 (0.11)	7.16 (0.13)	
		Expected success	7.65 (0.10)	7.45 (0.14)	

#### Expected Success

There were no significant main effects of candidate gender, *F*(1,96) = 1.27, *p* = 0.263, η^2^ = 0.01, participant gender *F*(1,96) = 2.34, *p* = 0.129, η^2^ = 0.02, or leadership characteristic *F*(1,96) = 2.57, *p* = 0.112, η^2^ = 0.03 (which does not support Hypothesis 1). There Candidate Gender × Participant Gender interaction effect was not significant, *F*(1,96) = 3.10, *p* = 0.082, η^2^ = 0.03, and a non-significant Leadership Characteristic × Participant Gender interaction, *F*(1,96) < 0.001, *p* = 0.995, η^2^ < 0.01. There was a significant Leadership Characteristic × Candidate Gender interaction, *F*(1,96) = 4.28, *p* = 0.041, η^2^ = 0.04. Consistent with Hypothesis 2, there was a preference for leadership potential over leadership performance for male candidates only, *F*(1,96) = 5.12, *p* = 0.026, η^2^ = 0.05, see Table 1, but not for female candidates, *F*(1,96) = 0.001, *p* = 0.981, η^2^ < 0.001. There was no significant differentiation between male and female leadership potential candidates, *F*(1,96) = 0.52, *p* = 0.473, η^2^ = 0.05, or between male and female leadership performance candidates, *F*(1,96) = 3.56, *p* = 0.06, η^2^ = 0.04.

Our exploratory analysis for participant gender revealed a significant Leadership Characteristic × Candidate Gender × Participant Gender interaction, *F*(1,96) = 5.85, *p* = 0.017, η^2^ = 0.06. We decomposed the three-way interaction by participant gender. Simple interactions showed that the Leadership Characteristic × Candidate Gender interaction was only significant among female participants, *F*(1,96) = 8.37, *p* = 0.005, η^2^ = 0.08, not among male participants, *F*(1,96) = 0.08, *p* = 0.783, η^2^ < 0.01.

The second order simple effect was significant among female participants who differentiated between candidates with leadership performance, *F*(1,96) = 7.94, *p* = 0.006, η^2^ = 0.08. Table 1 shows that female participants expected the female candidate with leadership performance to be more successful than the male candidate with leadership performance. Moreover, female participants expected the male candidate with leadership potential to be more successful than the male candidate with leadership performance, see Table 1, *F*(1,96) = 5.32, *p* = 0.023, η^2^ = 0.05. Female participants did not differentiate significantly between female candidates based on leadership characteristic, *F*(1,96) = 1.15, *p* = 0.287, η^2^ = 0.01, or between male and female candidates with leadership potential, *F*(1,96) = 0.54, *p* = 0.465, η^2^ < 0.01.

#### Résumé Evaluation

A Friedman test showed that the ranking evaluations of each candidate résumé were different, χ^2^(3) = 88.51, *p* < 0.001, see Table 2 for mean ranks. Wilcoxon signed rank tests provided support for our hypotheses. Specifically, male candidates with leadership potential were ranked higher than male candidates with leadership performance, *Z* = −6.36, *p* < 0.001 (Hypothesis 3). In contrast, female candidates with leadership performance were ranked higher than female candidates with leadership potential, *Z* = −4.70, *p* < 0.001 (Hypothesis 4). Furthermore, male candidates with leadership potential were ranked higher than female candidates with leadership potential, *Z* = −6.27, *p* < 0.001 (Hypothesis 5). Moreover, female candidates with leadership performance were ranked higher than male candidates with leadership performance, *Z* = −5.92, *p* < 0.001. In brief, in support of our hypotheses, male candidates with leadership potential were ranked as more impressive than male candidates with leadership performance. In contrast, female candidates with leadership performance were ranked as more impressive than female candidates with leadership potential.^3^

**Table 2:** Mean rank for each candidate for résumé evaluation and future performance (Experiment 1).

Candidate	Résumé evaluation	Future performance
Male leadership potential	1.74	1.80
Male leadership performance	3.30	3.24
Female leadership potential	2.87	2.86
Female leadership performance	2.09	2.10

#### Future Performance

A Friedman test showed that the rankings reflecting expectations of each candidate’s future performance were different, χ^2^(3) = 78.59, *p* < 0.001, see Table 2 for mean ranks. Wilcoxon signed rank tests revealed that male candidates with leadership potential were ranked higher than those candidates with leadership performance, *Z* = −6.12, *p* < 0.001 (Hypothesis 3). In contrast, female candidates with leadership performance were ranked higher than those with leadership potential, *Z* = −4.65, *p* < 0.001 (Hypothesis 4). Furthermore, male candidates with leadership potential were ranked higher than female candidates with leadership potential, *Z* = −6.00, *p* < 0.001 (Hypothesis 5). Finally, female candidates with leadership performance were ranked higher than male candidates with leadership performance, *Z* = −5.93, *p* < 0.001. In brief, results supported our hypotheses, with male candidates with leadership potential ranked more highly than those with leadership performance, but that this would not be the case for female candidates. Indeed, female candidates with leadership performance were ranked higher than female candidates with leadership potential.

### Discussion

Experiment 1 provides the first experimental evidence that female and male candidates’ leadership potential and leadership performance are evaluated differently. We did not find evidence for Hypothesis 1, an overall preference for potential. In line with an overlooked potential pattern, we found that participants expected male candidates with leadership potential to be more successful than male candidates with leadership performance (Hypothesis 3), although this was not the case for the candidate hiring measure. When participants ranked female candidates, they preferred leadership performance over leadership potential consistently across measures (support for Hypothesis 4). Interestingly, when participants were asked to rank candidates in evaluation of résumés and on future performance, female candidates’ leadership performance was preferred over that of male candidates. This type of ranking decision closely matches actual hiring processes, where final choices rely on rule-based selection criteria (e.g., ranking based on résumé evaluation).

We did not hypothesize effects of participant gender, but exploratory analysis revealed some differences. Specifically, a three-way interaction on candidates’ expected success showed that the two-way interaction was only significant among female participants. When judging candidates’ expected success, female participants rated female candidates with leadership performance as likely to be more successful than male candidates with leadership performance. Female participants also expected male candidates with leadership potential to be more successful than male candidates with leadership performance.

In this study, female candidates were rated as more hirable than male candidates. This unexpected finding is in line with a recent meta-analysis which showed that women are rated more effective than men in senior levels (Paustian-Underdahl et al., 2014). The stimulus materials presented to participants in Experiment 1 did not specify the level of leadership being recruited for. The information implied that the role was a relatively junior leadership position. This scenario had reasonable face validity because many fast-track programs are specifically designed to develop the potential of emerging talent (Singh et al., 2009; Thomas, 2009; Dries and Pepermans, 2012; Guan et al., 2014). Moreover, the principal motivation behind identifying leadership potential is to generate a pipeline of future leaders, which has major benefits (e.g., Williams-Lee, 2008; Poehlman and Newman, 2014). Nonetheless, the use of leadership potential as a selection criterion may be more common in the case of explicitly senior positions because many of the assessment tools used for selecting senior executives are related closely to those used to gauge high potential (Grabner and Moers, 2013). In Experiment 2, as well as retesting the overlooked potential effect, we therefore modified materials to highlight that the candidates were being considered for senior leadership positions. We also bolstered the measurement of the evaluation of expected success by using a more reliable multi-item measure. We also recruited a larger sample of participants. Finally, to provide a more direct test of Hypotheses 3-5, we asked participants to explicitly rank whom they would hire for the job.

## Experiment 2

### Materials and Methods

#### Participants and Design

Participants (*N* = 199; 126 females, 73 male *M*_age_ = 35.02, 78.4% in full or part-time employment) were recruited via an international online database, *Amazon MTurk*. The quasi experiment was a Leadership Characteristic × Candidate Gender × Participant Gender mixed design, with leadership characteristic and candidate gender as within-participant factors. All participants were exposed to a total of four résumés manipulating leadership characteristic (leadership potential and leadership performance) and candidate gender (male and female). To ensure consistency in other relevant résumé information, participants randomly received counterbalanced combinations of additional background information for each candidate.

#### Procedure and Materials

Individuals were invited, via an online platform, *Qualtrics*, to take part in a study on organizational decision-making. The experiment consisted of two phases. Participants were presented with an imitation Business News article describing the announcement of the retirement, and subsequent search for replacement, of the Director of Financial Affairs of a fictitious company, Tell Inc. The article provided background information about the organization, and a brief description that described Tell Inc.’s role as a growing and successful telecommunications company:

“In an open letter to Tell Inc. employees the CEO, Robin Metcalfe, announced the resignation of the company’s Vice President of Financial Affairs, Alex Hepburn, adding ‘Alex has been a great asset to this company having immeasurably contributed to our progress over recent years.’

Tell Inc. is a highly successful United States based telecommunications company, consistently performing well on the global markets, with particular growth and expansion in Eastern Europe and China over the last year. Tell Inc. is well known for its dynamic and innovative approach to communication technology, having developed some of the most well-known products on the market today.

This is a very important role for Tell Inc. to fill and there will be significant interest in the technology community about who will be appointed and which direction they will look to take the company in.

CEO Robin Metcalfe, said that they are looking to find ‘the best possible candidate to help lead and shape the bright future of Tell Inc.

All eyes are on the CEO and Board of Directors to see who they choose.”

Next, participants were presented each résumé (male leadership potential, female leadership potential, male leadership performance, female leadership performance). The background information and leadership scores (future leadership potential and previous leadership achievement) were the same as shown in Experiment 1. In Experiment 2, the résumés were made relevant to the hiring of a more senior candidate by changing candidates’ previous work experience to include at least one well known tech or communications company and by providing reviews from other people (previous employer and Tell Inc. CEO) and self-descriptions by the candidate. These comments reinforced either the candidates’ future leadership potential or previous leadership performance. The following examples show quotes from a CEO about a female candidate with leadership performance and about a male candidate with leadership potential, respectively:

“Christine is clearly a candidate who has performed very highly throughout her career. She has shown from her past achievements and accomplishments that she is highly capable of performing to the highest standard. Christine is certainly at the top of her group in her professional achievements.”

“Rupert is clearly a candidate who has shown excellent potential throughout his career. You can see from his budding talent and promise that he is highly capable of being one of the best in his field. Rupert is absolutely at the top of his vocation in terms of his professional potential.”

Participants then completed the evaluative rating measures (candidate hiring, expected success), immediately after reviewing each candidate. Next, all four résumés were presented simultaneously, so that participants could refresh their memory, and to minimize availability bias toward the most recently reviewed résumé. Participants then completed the dependent measures.

### Measures

#### Candidate Hiring

Candidate hiring was measured using two items (α = 0.85): “I would hire this candidate” and “this candidate would be a good appointment.” Items were measured on a rating scale and ranged from 1 (*strongly disagree*) to 9 (*strongly agree*).

#### Expected Success

Expected success was measured using six items to examine career and job success on a rating scale, from 1 (*strongly disagree*) to 9 (*strongly agree*) (α = 0.94; adapted from Ironson et al., 1989; Kossek et al., 2001). Items included: “How successful do you think each applicant will be in their career?”; “How successful do you think each applicant will be in their career, compared to other people?”; and “How successful do you think each applicant will be in their career, compared to the applicants’ significant others?”

#### Résumé Evaluation

Résumé evaluation was indicated by a choice of candidates, participants were asked “in your opinion, which applicant has the most impressive résumé?”(*first, second, third, fourth*), with first the most impressive.^4^

#### Future Performance

Future performance was assessed with the rank of candidates in response to the question “which candidate do you think will perform better by the fifth year?” (*first, second, third, fourth*), with *first* most likely to perform best.

#### Hire Choice

Hire choice was measured by participants rank choice of “which applicant would you hire?,” *first* to *fourth*, with *first* the choice of hire.

### Results

A Leadership Characteristic (leadership potential and leadership performance) × Candidate Gender (female and male) × Participant Gender (female and male) mixed ANOVA was used to analyze the measures of candidate hiring and expected success. As in Experiment 1, we hypothesized that participants would be more likely to hire candidates with leadership potential and would expect those candidates to be more successful than candidates with leadership performance (Hypothesis 1). Furthermore, we anticipated that these effects should be stronger for male candidates (Hypothesis 2). We did not hypothesize participant gender effects but included this factor as exploratory.

Friedman tests and Wilcoxon Signed Ranks tests were used to analyze whether there were differences in the choice-based rankings reflecting evaluations of each candidate’s résumé, future performance, and hire choice. Specifically, we expected participants to rank the male candidate with leadership potential higher the than male candidate with leadership performance on the evaluation of their résumés, future performance, and hire choice (Hypothesis 3). We predicted the opposite pattern for female candidates (Hypothesis 4). Finally, we expected participants to rank the male candidate with leadership potential higher than the female candidate with leadership potential in all ranking measures (Hypothesis 5).

#### Candidate Hiring

There was a significant effect of leadership characteristic, *F*(1,197) = 15.05, *p* < 0.001, η^2^ = 0.07. Participants rated candidates who exhibited leadership performance on their résumé more favorably than candidates who displayed leadership potential (see Table 3). This does not support Hypothesis 1. There was a near significant effect of participant gender, *F*(1,197) = 3.80, *p* = 0.053, η^2^ = 0.02. Table 3 shows that female participants rated candidates more highly than male participants. Contrary to Hypothesis 2, the Leadership Characteristic × Candidate Gender interaction was not significant, *F*(1,197) = 3.14, *p* = 0.078, η^2^ = 0.02. All remaining effects were not significant (see Table 4).

**Table 3:** Means and standard errors by candidate gender, participant gender, and leadership characteristic for candidate hiring and expected success (Experiment 2).

	Participant gender	Dependent measure	Leadership potential	Leadership performance	Overall
Female candidate	Female	Candidate hiring	7.16 (0.15)	7.68 (0.13)	7.42 (0.11)
		Expected success	7.47 (0.11)	7.84 (0.10)	7.65 (0.09)
	Male	Candidate hiring	6.78 (0.20)	7.39 (0.17)	7.09 (0.15)
		Expected success	7.33 (0.15)	7.60 (0.13)	7.47 (0.12)
	Overall	Candidate hiring	6.97 (0.13)	7.54 (0.11)	7.25 (0.09)
		Expected success	7.40 (0.09)	7.72 (0.08)	7.56 (0.08)
Male candidate	Female	Candidate hiring	7.41 (0.15)	7.57 (0.14)	7.49 (0.12)
		Expected success	7.78 (0.10)	7.80 (0.10)	7.79 (0.09)
	Male	Candidate hiring	7.03 (0.20)	7.33 (0.18)	7.18 (0.15)
		Expected success	7.36 (0.13)	7.73 (0.13)	7.55 (0.11)
	Overall	Candidate hiring	7.22 (0.12)	7.45 (0.11)	7.33 (0.10)
		Expected success	7.57 (0.08)	7.77 (0.08)	7.67 (0.07)
Candidate gender collapsed	Female	Candidate hiring	7.29 (0.13)	7.63 (0.11)	7.46 (0.10)
		Expected success	7.62 (0.09)	7.82 (0.08)	7.72 (0.08)
	Male	Candidate hiring	6.90 (0.17)	7.36 (0.14)	7.13 (0.13)
		Expected success	7.35 (0.12)	7.67 (0.11)	7.51 (0.10)
	Overall	Candidate hiring	7.10 (0.11)	7.49 (0.09)	
		Expected success	7.48 (0.08)	7.74 (0.07)	

#### Expected Success

There was a significant effect of leadership characteristic, *F*(1,197) = 17.72, *p* < 0.001, η^2^ = 0.08. Candidates with leadership performance were rated as more likely to be successful than those with leadership potential (Table 3); this does not support Hypothesis 1. All other main effects and two-way interactions were not significant (see Table 4).

**Table 4:** ANOVA summary statistics (Experiment 2).

	Candidate hiring	Expected success
Leader characteristic (LC)	15.05, *p* < 0.001, η^2^ = 0.07	17.72, *p* < 0.001, η^2^ = 0.08
Candidate gender (CG)	0.82, *p* = 0.365, η^2^ < 0.001	2.65, *p* = 0.105, η^2^ = 0.01
Participant gender (PG)	3.80, *p* = 0.053, η^2^ = 0.02	2.71, *p* = 0.101, η^2^ = 0.01
LC × CG	3.14, *p* = 0.078, η^2^ = 0.02	1.08, *p* = 0.299, η^2^ = 0.01
LC × PG	0.32, *p* = 0.571, η^2^ < 0.001	1.01, *p* = 0.105, η^2^ = 0.01
CG × PG	0.02, *p* = 0.897, η^2^ < 0.001	0.18, *p* = 0.669, η^2^ = 0.001
LC × CG × PG	0.03, *p* = 0.874, η^2^ < 0.001	3.79, *p* = 0.053, η^2^ = 0.02

*F(1,197)*

There was a near significant Leadership Characteristic × Candidate Gender × Participant Gender interaction, *F*(1,197) = 3.79, *p* = 0.053, η^2^ = 0.02. We decomposed the three-way interaction by participant gender. Simple interaction effects showed that the Leadership Characteristic × Candidate Gender interaction was only significant for female participants, *F*(1,197) = 6.08, *p* = 0.015, η^2^ = 0.03, and not for male participants, *F*(1,197) = 0.32, *p* = 0.571, η^2^ = 0.002. Second order simple effects show that female participants expected the female candidate with leadership performance to be more successful than the female candidate with leadership potential, *F*(1,197) = 12.15, *p* = 0.001, η^2^ = 0.06. In addition, Table 3 shows that the female participants expected the male candidate with leadership potential to be more successful than the female candidate with leadership potential, *F*(1,197) = 9.12, *p* = 0.003, η^2^ = 0.04. Female participants did not differentiate significantly between the male candidates based on leadership characteristic, *F*(1,197) = 0.04, *p* = 0.842, η^2^ < 0.001, or between male and female candidates with leadership performance, *F*(1,197) = 0.15, *p* = 0.703, η^2^ = 0.001.

#### Résumé Evaluation

A Friedman test showed that the rankings of the résumés differed, χ^2^(3) = 185.25, *p* < 0.001. Wilcoxon signed rank tests supported our hypotheses, and Table 5 shows the mean rank per candidate. Male candidates with leadership potential were ranked more highly than male candidates with leadership performance, *Z* = −9.79, *p* < 0.001 (Hypothesis 3). In contrast, female candidates with leadership performance were ranked more highly than female candidates with leadership potential, *Z* = −6.19, *p* < 0.001 (Hypothesis 4). Furthermore, male candidates with leadership potential were ranked more highly than female candidates with leadership potential, *Z* = −9.76, *p* < 0.001 (Hypothesis 5). Finally, female candidates with leadership performance were ranked more highly than male candidates with leadership performance, *Z* = −7.61, *p* < 0.001.^5^

**Table 5:** Mean rank for each candidate for résumé evaluation, future performance, and hire choice (Experiment 2).

Candidate	Résumé evaluation	Future performance	Hire choice
Male leadership potential	1.64	1.80	1.75
Male leadership performance	3.25	3.22	3.32
Female leadership potential	2.90	2.73	2.74
Female leadership performance	2.20	2.25	2.20

#### Future Performance

A Friedman test showed that the four candidates’ future performances were ranked differently, χ^2^(3) = 133.85, *p* < 0.001. Wilcoxon signed rank tests supported our hypotheses, and Table 5 shows the mean rank per candidate. The future performance of male candidates with leadership potential was ranked more highly than that of male candidates with leadership performance, *Z* = −8.71, *p* < 0.001 (Hypothesis 3). In contrast, the future performance of female candidates with leadership performance was ranked more highly than that of female candidates with leadership potential, *Z* = −3.80, *p* < 0.001 (Hypothesis 4). Furthermore, male candidates with leadership potential were ranked more highly than female candidates with leadership potential, *Z* = −7.65, *p* < 0.001 (Hypothesis 5). Finally, female candidates with leadership performance were ranked more highly than male candidates with leadership performance, *Z* = −8.05, *p* < 0.001.

#### Hire Choice

A Friedman test showed that hiring preference differed among the four candidates, χ^2^(3) = 164.84, *p* < 0.001. Wilcoxon signed rank tests supported our hypotheses, and Table 5 shows the mean rank per candidate. Specifically, male candidates with leadership potential were more likely to be the hire than those with leadership performance *Z* = −9.56, *p* < 0.001 (Hypothesis 3). In contrast, female candidates with leadership performance were more likely to be the hire than those with leadership potential, *Z* = −4.36, *p* < 0.001 (Hypothesis 4). Furthermore, male candidates with leadership potential were more likely to be the hire than female candidates with leadership potential, *Z* = −8.44, *p* < 0.001 (Hypothesis 5). Finally, female candidates with leadership performance were more likely to be the hire than male candidates with leadership performance, *Z* = −8.42, *p* < 0.001.

### Discussion

Experiment 2 provides evidence that candidates’ gender moderates evaluations of their leadership characteristics. Consistent findings across the ranking measures provide clear evidence regarding the overlooked potential effect. We found that when participants ranked male candidates there was a preference for potential (Hypothesis 3), whereas leadership potential was overlooked when they ranked female candidates (Hypotheses 4 and 5). Indeed, consistent with Experiment 1, when participants judged female candidates, leadership performance was preferred over leadership potential. Moreover, the finding that leadership potential led to an upgrading of (otherwise equivalent) male candidates relative to female candidates, and that leadership performance led to a downgrading of male relative to female candidates seems highly consistent with the interpretation that gender role expectations moderated judgments of the candidates.

In our exploratory analysis we also found some evidence that participant gender affected these judgments. Specifically, an interaction between candidate gender and leadership characteristic on expectations about candidates’ success was significant among female participants but not male participants. Female participants rated the male candidate with leadership potential higher than the female candidate with leadership potential. Additionally, female participants expected the female candidate with leadership performance to be more successful than the female candidate with leadership potential.

## General Discussion

Our findings provide several new empirical and theoretical contributions. Overall, these studies provide the first experimental evidence that a candidate’s gender can affect evaluators’ assessment of the value of their leadership potential and leadership performance. In both experiments, consistent with our Hypotheses 3, 4, and 5, leadership potential was preferred when participants ranked male candidates, whereas potential was overlooked when participants ranked female candidates. Male candidates that demonstrated higher potential were perceived to have a more impressive résumé and were expected to perform better in the future than male candidates who demonstrated higher performance. In contrast, female candidates who demonstrated higher performance were perceived to have a more impressive résumé and were expected to perform better than female candidates who demonstrated higher potential. If these findings were extrapolated to real hiring situations, they would mean that whilst women’s past performance would have to at least as good as men’s, women would be held to higher standards in selection processes because their leadership potential would be less likely to be recognized than men’s. The findings emerged most clearly when participants ranked rather than rated candidates. The ranking data are likely to have higher ecological validity given that most recruitment procedures conclude with a ranking process in order to decide which candidate to hire.

Why might men with future leadership potential have a distinctive advantage? One explanation can be drawn from role incongruity theory which highlights that people have a powerful implicit association between leadership and agentic traits (Eagly and Karau, 2002; Heilman and Eagly, 2008). Female candidates who foreground their leadership potential may challenge people’s expectations about how women in leadership positions should behave, thereby highlighting role incongruence. Therefore, they may be subjected to greater discrimination than women who primarily emphasize their past leadership performance. The current data do not allow us to test this possibility directly, and further work will be needed to explore this further.

We explored the impact of gender on preference for leadership potential and/or leadership performance. On candidate choice rankings an unexpected but consistent finding was that participants prioritized female’s leadership performance over that of male candidates. It could be that women are implicitly required to show greater evidence of competence to overcome stereotypically negative performance expectations, particularly in male gender-typed job domains (Lyness and Heilman, 2006). Therefore, women are more likely to have to demonstrate a successful background in order to show congruence between their skills and the leadership position, and to overcome role-congruity bias (Koch et al., 2015).

Despite generally consistent findings, a few inconsistencies merit discussion. These may reflect that the two studies assessed judgments relating to different levels of seniority (higher in Experiment 2). In Experiment 1, but not in Experiment 2, participants perceived female candidates to be a better hire overall. This unexpected result might have been driven by participants’ reactions to encountering counterstereotypical high-performing women, an advantage that may be worth exploring in case it is limited to judgments about junior leadership roles.

There was also some evidence of gender ingroup bias in both experiments but it was not ubiquitous. Although ingroup bias is a robust social psychological phenomenon (Hewstone et al., 2002), particularly amongst members of more dominant and socially valued groups (Rudman et al., 2002), gender is sometimes an exception to this pattern. This exception is because the more dominant group (i.e., men) are less likely to show direct ingroup bias (Rudman and Goodwin, 2004) perhaps owing to more subtle forms of prejudice (Glick and Fiske, 2001). In Experiment 1, female participants showed ingroup bias in their evaluations of the candidates. In Experiment 2, only female participants demonstrated differences in evaluations of expected success for female candidates based on their potential or performance. This finding suggests further nuanced differences between leadership potential and leadership performance which are likely intrinsically linked to perceptions of gender and leadership. These difference warrant further attention in follow-up research, as they suggest that the demonstration of leadership potential (vs. performance) could also be based in gender role expectations, like ambition.

Going beyond previous research, these studies demonstrated that when faced with a choice, judges consistently ranked male candidates with leadership potential over their female counterparts. Our ranking findings are of particular significance as they mirror the majority of selection and recruitment decisions, whereby only one candidate can be offered the job. Moreover, processes that identify and fast-track leadership potential are already in place in many organizations (e.g., McDonnell et al., 2010). Understanding how gender might influence the perception, promotion, and development of leadership potential over time and career is vital in promoting equality. This research illustrates, for the first time, a subtle but powerful way in which women are discriminated against in the workplace as a direct result of their gender.

### Limitations and Future Directions

We have provided evidence that leadership potential and leadership performance can yield different hiring and evaluation outcomes for men and women. Various limitations need to be considered before making strong conclusions. First, the extant literature lacks a well-developed empirical foundation for the theoretical distinction between leadership potential and leadership performance. We therefore relied on a general definition of leadership potential, which might not fully encompass the entire array of traits linked to leadership potential or their relationship with leadership performance. We chose to focus distinctly on either past performance or future potential to avoid confusion. We gave the manipulations context and reinforcement in Experiment 2 by providing a richer view of the candidate (e.g., using assessments from previous and prospective employers). Overall, even if a more comprehensive basis for the distinction could improve the design of descriptions in vignettes, the results do show that participants responded differently to the leadership potential and leadership performance depictions of candidates.

We used a crowdsourcing platform which had the advantage of using a real-world sample of employed people across a range of occupations. Nonetheless, it is possible that this approach also introduced more unexplained variability in the sample (e.g., variability in organizational culture) than might be attained with a more homogenous sample (e.g., based in a single organization). Future research could investigate the generality of the overlooked potential effect in single organizational contexts, or compare different organizational contexts that are more typically male- or female-dominated. Moreover, it is conceivable that differences amongst participants’ own occupancy of leadership positions, may have influenced their responses. Future research should investigate potential moderating effects of participant leadership experience. A further way to pursue future studies would be to test the effect using samples of hiring managers and members of promotion panels.

Additional directions for research include investigating boundary conditions for the effect such as different leadership goals (e.g., more task-oriented or socio-emotional), or culture. For example, high potential women are regarded as having higher diversity value (Leslie et al., 2017). It would be interesting to test the overlooked potential effect in contexts where diversity goals are salient or not. Using diversity as a boundary condition could also open potential avenues to future interventions.

The degree of role incongruity could also be pursued as a moderating factor. A further subtlety may be that the linguistic framing of the role positions may affect whether or not potential is overlooked. For example, Horvath and Sczesny (2015) found that female and male candidates for a high status leadership position were perceived as fitting equally well to the job when the job advert used feminine-masculine word pairs (instead of solely masculine forms). Linguistic framing might also be relevant for the overlooked potential effect.

Real hiring decisions are based on choices, which our ranking measures simulated. However, the decision to use ranking measures imposed limits to our capacity to investigate moderation effects. Moreover, hiring decisions are often based not only on résumé evaluations but also subsequent rounds of interviews. The present research only speaks to the first stage of this selection process. It may be that these interviews either ameliorate or exacerbate the overlooked potential effect, which also warrants investigation in future research.

## Conclusion and Implications

The present research has practical implications for organizations, and possibly even beyond. For example, if preference for leadership potential in men is a generic phenomenon, it may well confer unfair advantages well beyond commercial and business contexts (e.g., in education, politics, journalism, the legal system). For any organization, ensuring that hiring processes are fair and offer equal opportunities is fundamental for attaining gender equity in leadership positions. Given that employers typically regard leadership potential as a desirable trait (Church, 2014), raising awareness that potential is likely to be undervalued when people judge women may offer a method to improve diversity and equality in leadership. Previous evidence has found that there can be a preference for leadership potential (Tormala et al., 2012), our research highlights that this may be an advantage from which men alone benefit. Our research suggests that women’s prospects seem to rest more exclusively on their demonstration of leadership performance over potential. Potential implies that an individual has the quality to perform in wider or different roles in the organization at a later stage (Silzer and Church, 2009). If higher potential among women is not recognized, women may find they are trapped in particular silos (such as administration or human resources), and are at a disadvantage when it comes to more overarching roles and positions. By not fully recognizing leadership potential in female candidates, organizations are inhibiting the prospects of half of their talent. This inhibition ironically means organizations may be less likely to achieve their own full potential.

## Ethics Statement

All experiments were carried out in accordance with the recommendations of the School of Psychology Ethics Committee at the University of Kent, United Kingdom. The protocol was approved by the School of Psychology Ethics process. All participants gave written informed consent in accordance with the Declaration of Helsinki. The research was conducted in accordance with guidelines from the University of Kent Research Ethics (Human Participants) Committee, the Economic and Social Research Council (ESRC) Research Ethics Framework, and the ethical guidelines from the British Psychology Society (BPS).

## Author Contributions

AP and GR conceived of the presented idea and took the joint lead in writing the manuscript. AL, DA, and FT contributed in theory development. AL and DA bolstered analytical methods. GR and DA encouraged AP to pursue the application of leadership potential and gender to leadership selection and supervised the findings of this research. DA worked on revisions for the final version. All authors provided critical feedback and helped to shape the overall research.

## Conflict of Interest Statement

The authors declare that the research was conducted in the absence of any commercial or financial relationships that could be construed as a potential conflict of interest.

## Footnotes

1 This was identical to Tormala et al. (2012, Experiment 2) including the typographical error “the applicant predicted future” rather than “the applicant’s predicted future.”

2 We also asked participants to make a choice of résumé based on the following item “at present, which applicant had a more objectively impressive résumé?” Results were the same for this measure, and given that the items are very similar and taken that ranking data cannot be aggregated into an average score, we opted to report the results for the first measure. Results for the second item are available from the corresponding author on request.

3 Given the ranking nature of the data we were not able to test for interactions with participant gender. However, we conducted Friedman tests, and Wilcoxon signed rank tests, separately for female and male participants for the measures of evaluation of résumé and future performance. The pattern of results was identical for each participant gender group. Please refer to Supplemental Materials for the results of these exploratory analyses.

4 Similar to Experiment 1, we also asked participants to compare résumés based on the following item “at present, which applicant had a more objectively impressive résumé?” Results were the same for this measure, and given that the items are very similar and ranking data cannot be aggregated into an average score, we opted to report the results for the first measure. Results for the second item are available from the corresponding author on request.

5 Similarly to Experiment 1, we conducted Friedman tests and Wilcoxon signed rank tests separately for female and male participants for all ranking measures. The pattern of results was identical for each participant gender group, with two exceptions. Specifically, for the measure of future performance and hire choice, male participants ranked the female candidate with leadership potential similarly to the female candidate with leadership performance. However, as hypothesized, both female and male participants ranked the male candidate with leadership potential higher than the male candidate with leadership performance. Please refer to Supplemental Materials for the results of these exploratory analyses.

## Appendix: Manipulations Experiment 1

**Table:**

Applicant A	Applicant B	Applicant C	Applicant D
Sex: Male	Sex: Female	Sex: Female	Sex: Male
Birthday: 09/21/1986	Birthday: 05/13/1987	Birthday: 30/08/1985	Birthday: 19/12/1984
**Educational Background:**	**Educational Background:**	**Educational Background:**	**Educational Background:**
B.A., 2007, Cornell University	B.A., 2008, University of California, Berkeley	B.A., 2006, Brown University	B.A., 2005, University of Notre Dame
Major: Accounting, GPA: 3.82	Major: Economics, GPA: 3.90	Major: Management Accountancy, GPA: 3.79	Major: Finance, GPA: 3.91
M.B.A., 2011, New York University	M.S., 2011, Management Science, UCLA	M.B.A., 2009, University of Washington	M.S., 2008, Operational Research and Management Science, University of Michigan
**Internships:**	**Internships:**	**Internships:**	**Internships:**
Ernst and Young	Morgan Stanley	Merrill Lynch	American Express
Morgan Stanley	Fidelity Investments	Susquehanna International Group	General Electric
**Panel Review:**	**Panel Review:**	**Panel Review:**	**Panel Review:**
“This applicant has a budding career in front of him. He has clearly demonstrated some highly valuable attributes that would make significant contributions to this organization. Great potential!”	“This candidate has an excellent track-record, she has consistently achieved to a high standard. In addition she has made significant contributions to the performance of the company by exceeding her targets.”	“This candidate has great prospects. She has some exciting new ideas for the future of the team and the organization, which could offer the opportunity to increase sales and performance in the future.”	“The applicant is highly capable, and has consistently performed above his own objectives and that of the organizations. The performance in his current role has exceeded expectations.”
**Job Testing:**	**Job Testing:**	**Job Testing:**	**Job Testing:**
83/100 on the Leadership Achievement Inventory (LAI)	96/100 on the Leadership Achievement Inventory (LAI)	84/100 on the Leadership Achievement Inventory (LAI)	94/100 on the Leadership Achievement Inventory (LAI)
96/100 on the Assessment of Leadership Potential (ALP)	83/100 on the Assessment of Leadership Potential (ALP)	94/100 on the Assessment of Leadership Potential (ALP)	84/100 on the Assessment of Leadership Potential (ALP)

*• The Leadership Achievement Inventory (LAI) gauges leadership achievement, defined as an individual’s observed (i.e., actual) leadership performance at the current stage in his or her career. An achievement score of 96 places this applicant in the top 4% of people who have been assessed. •The Assessment of Leadership Potential (ALP) gauges leadership potential, defined as the employee’s predicted leadership performance in the near future. A score of 83 indicates that this applicant predicted future leadership performance is estimated to be in the top 17% of people who have been assessed.*
