# Supplementary material for: Overlooked Leadership Potential: The Preference for Leadership Potential in Job Candidates Who Are Men vs. Women
Source: Front Psychol. 2019 Apr 16;10:755. doi: 10.3389/fpsyg.2019.00755 (PMC6476968; doi:10.3389/fpsyg.2019.00755)
Supplement: Supplementary file 1 [file Data_Sheet_1.pdf]

Supplemental Materials

Study 1 results on ranking measures for female and male participants

Résumé evaluation. We conducted Friedman tests separately for female and male participants to explore whether the rankings reflecting expectations of each candidate's impressiveness were similar irrespective of gender, or whether they differed. The analyses show that the mean ranks were equivalent for female participants, χ^2 (3) = 48.45, $p < .001$, and for male participants, χ^2 (3) = 43.66, $p < .001$ (see Table S1). Wilcoxon signed rank tests provided support for our hypotheses irrespective of gender. Specifically, male candidates with leadership potential were ranked higher than male candidates with leadership performance in terms of the evaluation of their résumés by female participants, $Z = -4.25$, $p < .001$, and male participants, $Z = -4.69$, $p < .001$. In contrast, female candidates with leadership performance were ranked higher than female candidates with leadership potential, by female participants, $Z = -3.85$, $p <.001$, and by male participants, $Z = -3.08$, $p = .002$. Furthermore, male candidates with leadership potential were ranked higher than female candidates with leadership potential, by female participants, $Z = -3.65$, $p < .001$, and by male participants, $Z = -5.22$, $p < .001$. Moreover, female candidates with leadership performance were ranked higher than male candidates with leadership performance, by female participants, $Z = -5.02$, $p < .001$, and by male participants, $Z = -3.58$, $p < .001$. In brief, results show that when making a choice between candidates, both female and male participants ranked the résumé of male candidates with leadership potential as more impressive than the résumé of male candidates with leadership performance. Female candidates' résumés were ranked as more impressive when the candidate had leadership performance rather than leadership potential, by female participants and by male participants.

Future performance. We conducted Friedman tests separately for female and male

participants to explore whether the rankings reflecting expectations of each candidate's future performance were similar irrespective of gender, or whether they differed. The analyses show that the mean ranks were equivalent for female participants, χ^2 (3) = 37.74, $p < .001$, and for male participants, χ^2 (3) = 42.64, $p < .001$ (see Table S1). Wilcoxon signed rank tests revealed that male candidates with leadership potential were ranked higher than those candidates with leadership performance, by female participants, $Z = -3.93$, $p < .001$, and by male participants, $Z = -4.65$, $p < .001$. In contrast, female candidates with leadership performance were ranked higher than those with leadership potential, by female participants, $Z = -3.35$, $p < .001$, and by male participants, $Z = -3.34$, $p = .001$. Furthermore, male candidates with leadership potential were ranked higher than female candidates with leadership potential, by female participants, $Z = -3.30$, $p = .001$, and by male participants, $Z = -5.10$, $p = .001$. Finally, female candidates with leadership performance were ranked higher than male candidates with leadership performance, by female participants, $Z = -4.74$, $p < .001$, and by male participants, $Z = -3.80$, $p < .001$. In brief, results show that both female and male participants ranked male candidates with leadership potential more highly than those with leadership performance, but that this effect did not emerge for female candidates. Indeed, female candidates with leadership performance were ranked higher than female candidates with leadership potential.

Study 2 results on ranking measures for female and male participants

Résumé evaluation. We conducted Friedman tests separately for female and male participants to explore whether the rankings reflecting expectations of each candidate's impressiveness were similar irrespective of gender, or whether they differed. The analyses show that the mean ranks were equivalent for female participants, χ^2 (3) = 145.15, $p < .001$, and for male participants, χ^2 (3) = 44.72, $p < .001$ (see Table S2). Wilcoxon signed rank tests provided support for our hypotheses irrespective of gender. Specifically, male candidates

with leadership potential were ranked higher than male candidates with leadership performance in terms of the evaluation of their résumés by female participants, $Z = -8.58$, $p < .001$, and by male participants, $Z = -4.78$, $p < .001$. In contrast, female candidates with leadership performance were ranked higher than female candidates with leadership potential, by female participants, $Z = -5.32$, $p < .001$, and by male participants, $Z = -3.29$, $p = .001$. Furthermore, male candidates with leadership potential were ranked higher than female candidates with leadership potential, by female participants, $Z = -7.90$, $p < .001$, and by male participants, $Z = -5.63$, $p < .001$. Moreover, female candidates with leadership performance were ranked higher than male candidates with leadership performance, by female participants, $Z = -6.70$, $p < .001$, and by male participants, $Z = -3.74$, $p < .001$. In brief, results show that when making a choice between candidates, both female and male participants ranked the résumé of male candidates with leadership potential as more impressive than the résumé of male candidates with leadership performance. Female candidates' résumés were ranked as more impressive when the candidate had leadership performance rather than leadership potential, by female participants and by male participants.

Future performance. We conducted Friedman tests separately for female and male participants to explore whether the rankings reflecting expectations of each candidate's future performance were similar irrespective of gender, or whether they differed. The analyses show that the mean ranks were equivalent for female participants, $\chi^2(3) = 107.69$, $p < .001$, and for male participants, $\chi^2(3) = 30.42$, $p < .001$ (see Table S2). Wilcoxon signed rank tests revealed that male candidates with leadership potential were ranked higher than those candidates with leadership performance, by female participants, $Z = -7.68$, $p < .001$, and by male participants, $Z = -4.25$, $p < .001$. In contrast, female candidates with leadership performance were ranked higher than those with leadership potential, but only by female participants, $Z = -3.86$, $p < .001$; not by male participants, $Z = -1.27$, $p = .203$. Furthermore,

male candidates with leadership potential were ranked higher than female candidates with leadership potential, by female participants, $Z = -7.10$, $p < .001$, and by male participants, $Z = -3.24$, $p = .001$. Finally, female candidates with leadership performance were ranked higher than male candidates with leadership performance, by female participants, $Z = -6.71$, $p < .001$, and by male participants, $Z = -4.34$, $p < .001$. In brief, results show that both female and male participants ranked male candidates with leadership potential more highly than those with leadership performance. However, only female participants ranked female candidates with leadership performance higher than female candidates with leadership potential.

Hire choice. We conducted Friedman tests separately for female and male participants to explore whether the rankings reflecting hiring preferences were similar irrespective of gender, or whether they differed. The analyses show that the mean ranks were equivalent for female participants, χ^2 (3) = 119.67, $p < .001$, and male participants, χ^2 (3) = 47.42, $p < .001$ (see Table S2). Wilcoxon signed rank tests revealed that male candidates with leadership potential were ranked higher than those candidates with leadership performance, by female participants $Z = -8.05$, $p < .001$, and by male participants, $Z = -5.25$, $p < .001$. In contrast, female candidates with leadership performance were ranked higher than those with leadership potential, but only by female participants, $Z = -4.28$, $p < .001$; not by male participants, $Z = -1.68$, $p = .093$. Furthermore, male candidates with leadership potential were ranked higher than female candidates with leadership potential, by female participants, $Z = -7.37$, $p < .001$, and by male participants, $Z = -4.16$, $p < .001$. Finally, female candidates with leadership performance were ranked higher than male candidates with leadership performance, by female participants, $Z = -6.85$, $p < .001$, and by male participants, $Z = -4.86$, $p < .001$. In brief, results show that both female and male participants ranked male candidates with leadership potential more highly than those with leadership performance, but that this effect only emerged for female candidates ranked by female participants.

Table S1

Mean rank for each candidate for résumé evaluation and future performance for female and male participants (Experiment 1).

	Female Participants		Male Participants	
Candidate	Résumé evaluation	Future performance	Résumé evaluation	Future performance
Male leadership potential	1.74	1.82	1.75	1.78
Male with performance	3.54	3.41	3.14	3.14
Female leadership potential	2.79	2.77	2.92	2.92
Female leadership performance	1.92	2.00	2.20	2.17

Table S2

Mean rank for each candidate for résumé evaluation, future performance, and hire choice for female and male participants (Experiment 2).

	Female Participants			Male Participants		
Candidate	Résumé evaluation	Future performance	Hire choice	Résumé evaluation	Future performance	Hire choice
Male leadership potential	1.55	1.71	1.68	1.81	1.97	1.86
Male leadership performance	3.37	3.29	3.34	3.05	3.11	3.27
Female leadership potential	2.90	2.80	2.81	2.90	2.60	2.62
Female leadership performance	2.18	2.21	2.17	2.23	2.32	2.25
